# CX1/BtSY2 and BANAL-20-52 exhibit broader receptor binding and higher affinities to multiple animal ACE2 orthologs than SARS-CoV-2 prototype

**DOI:** 10.1128/jvi.00283-25

**Published:** 2025-07-10

**Authors:** Zepeng Xu, Linjie Li, Yuhang Gu, Dedong Li, Jianxun Qi, Kefang Liu, Chu-Xia Deng, George Fu Gao

**Affiliations:** 1 Faculty of Health Sciences, University of Macau, Macau SAR, China; 2 Beijing Life Science Academy 679188 , Beijing, China; 3 CAS Key Laboratory of Pathogen Microbiology and Immunology, Institute of Microbiology, Chinese Academy of Sciences 85387, Beijing, China; 4 School of Life Sciences, Yunnan University 674423 , Kunming, China; The Ohio State University, Columbus, Ohio, USA

**Keywords:** SARS-CoV-2 related coronaviruses, host range, interspecies transmission, CX1, BANAL-20-52, human ACE2, spike, cryo-EM

## Abstract

Animal coronaviruses (CoVs) CX1 (formerly named BtSY2) and BANAL-20-52 are phylogenetically closely related to severe acute respiratory syndrome coronavirus 2 (SARS-CoV-2), and CX1 is the first observed animal betaCoV carrying naturally occurring Y501 in its receptor-binding domain (RBD) of the spike (S) protein, a residue related to human adaptation and broader host range. We evaluated the ACE2 usage of CX1 and BANAL-20-52 and observed broader receptor binding spectra and higher affinities to most of the tested animal ACE2 orthologs than the SARS-CoV-2 prototype. Determination of the cryo-EM structures of their S proteins and RBD/hACE2 complexes reveals that Y501 is inter-replaceable with H498 substitution while synergetic with R498 for human ACE2 binding. These results provide further structural insights into SARS-CoV-2 receptor recognition and address the importance of surveillance on potential emerging CoVs.

**IMPORTANCE:**

Since the outbreak of COVID-19, forewarning and prevention of the next pandemic have been widely discussed. Coronaviruses (CoVs) CX1 (formerly named BtSY2) and BANAL-20-52 are phylogenetically closely related to SARS-CoV-2. Particularly, CX1 is the first SARS-CoV-2-related CoV containing Y501 in its receptor-binding domain (RBD) of the spike (S) protein. This study evaluated the interspecies transmission potential of the two CoVs and structurally elucidated the interplay between two RBD residues 498 and 501 on ACE2 binding, further highlighting the importance of surveillance on zoonotic CoVs.

## INTRODUCTION

Since the outbreak of coronavirus disease 2019 (COVID-19), forewarning and prevention of the next pandemic have been widely discussed, and surveillance on animal coronaviruses (CoVs) with interspecies transmission potential is a crucial part of the early warning system (1–3). Severe acute respiratory syndrome coronavirus 2 (SARS-CoV-2) has been evolving even into new serotypes, further highlighting the importance of moving forward the preventing frontline from human to animals (4–7). Multiple related CoVs have been reported to have a wide potential host range, suggesting their risk of interspecies spillover (8–11). Moreover, occasional zoonotic spillover events of animal CoVs, sometimes with acute respiratory symptoms, have been reported in multiple countries, further highlighting the continuous risk of CoV interspecies transmission (12–14). Thus, constant surveillance on animal CoVs is still important for forewarning of the next pandemic.

Recently, CX1 (formerly named BtSY2), another SARS-CoV-2-related CoV from *Rhinolophus pusillus* and *Rhinolophus marshalli*, is reported (15). Particularly, CX1 spike (S) protein exhibits 98.0% amino acid (AA) identity to that of SARS-CoV-2 prototype (PT) (GISAID: EPI_ISL_402119), with only seven distinct residues on the receptor-binding domain (RBD), even less than some SARS-CoV-2 Omicron sub-variants to the PT. However, three of the seven RBD substitutions are located on the receptor angiotensin-converting enzyme 2 (ACE2)-binding interface (F486L, Q498H, N501Y), which are reported to affect human ACE2 (hACE2) binding and host tropism (16–19). Notably, CX1 is the first SARS-CoV-2-related CoV that carries Y501, an important substitution not only markedly enhancing hACE2 binding but also enabling host range expansion (20, 21). Moreover, N501Y is one of the earliest emerging substitutions after the COVID-19 outbreak and was fixed in nearly all the dominantly circulating SARS-CoV-2 variants/sub-variants, indicating its important role in human adaptation (20, 22). Another related CoV, BANAL-20-52, demonstrated the highest S protein identity to SARS-CoV-2 (98.4% AA identity). However, there is no reported structure of the complex of its S trimer or RBD binding with ACE2. Therefore, the molecular characteristics of both CX1 and BANAL-20-52 recognizing hACE2 need to be elucidated.

The receptor binding spectra of SARS-CoV-2 and its related CoVs have been a scientific hotspot, and several host range-determining residues have been identified by mounting investigations into structural analysis on ACE2 orthologs binding with RBDs from SARS-CoV-2 and its related CoVs (16, 18, 23–25). By December 2024, 56 species have been reported to be naturally infected by SARS-CoV-2 (26). Residues on 493, 498, and 501 in the S protein, among others, are reported as main determinants of potential host range (16, 18).

In this study, we evaluated the receptor usage of CX1 and BANAL-20-52 and observed broader potential receptor binding spectra than that of the PT, suggesting the risk of interspecies spillover. Determination and analysis of cryo-EM structures of their S proteins and RBD/hACE2 complexes revealed that Y501 shows a synergetic effect with R498 and inter-replaceability with H498 for hACE2 binding. These results provide a structural basis for CX1 and BANAL-20-52 interspecies transmission and address the importance of surveillance on potential emerging CoVs.

## RESULTS

### CX1 and BANAL-20-52 demonstrate efficient hACE2 usage

**Fig 1:**
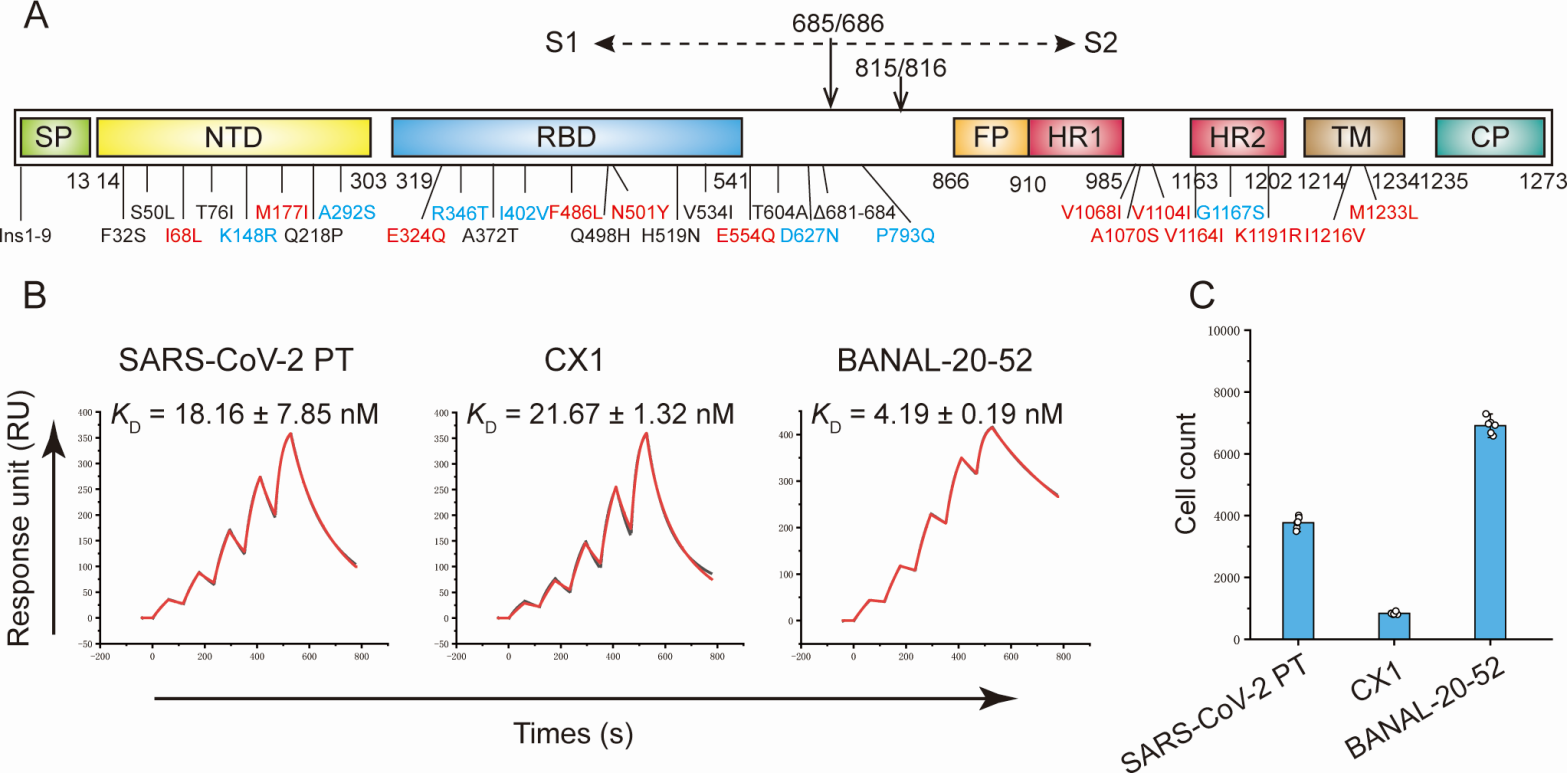
hACE2 usage of CX1 and BANAL-20-52. (**A**) Mutation mapping of CX1 and BANAL-20-52 S proteins. Distinctive residues from SARS-CoV-2 PT are labeled (numbering based on SARS-CoV-2 PT). Mutations shared by both CoVs are colored in black, and those exclusive to CX1 and BANAL-20-52 are colored in red and blue, respectively. (**B**) The SPR assay of SARS-CoV-2 PT, CX1, and BANAL-20-52. Black and red lines represent the actual and fitted curves. The *K*_D_ is presented as mean ± standard deviation (SD) from three independent repeats. (**C**) Pseudovirus assay of SARS-CoV-2 PT, CX1, and BANAL-20-52. The assay was conducted on Vero cells. Data were presented as mean ± SD. Two independent experiments were performed with similar results.

The S protein sequences of both CX1 and BANAL-20-52 are closely related to SARS-CoV-2 PT S protein and share high AA identity (97.96% and 98.43%, respectively) (Fig. 1A; Fig. S1). Sequence alignment demonstrates that both CX1 and BANAL-20-52 carry Threonine (T) on site 372, which is reported to play a pivotal role in the infectivity of SARS-CoV-2 (9, 27). Additionally, both S proteins lack the PRRA insertion on site 681, resulting in the absence of furin-cleavage site (FCS) facilitating S1 subunit dissociation (Fig. S2).

To evaluate the receptor usage of CX1 and BANAL-20-52 S proteins, surface plasmon resonance (SPR) as well as pseudovirus infection assay was performed. The SPR results show that CX1-RBD binds to hACE2 with a comparable affinity to SARS-CoV-2 PT-RBD with the dissociation constant (*K*_D_) of 21.67 nM vs 18.16 nM, whereas BANAL-20-52-RBD demonstrates 4.3-fold higher affinity than the PT (4.19 nM vs 18.16 nM) (Fig. 1B). To further validate the hACE2 usage, we then conducted a pseudovirus assay. The S proteins of SARS-CoV-2, CX1, and BANAL-20-52 were incorporated into pseudovirions with comparable efficiency (Fig. S3). Meanwhile, full-length S proteins of CX1 and BANAL-20-52 (~180 kD) were observed, consistent with the absence of FCS. CX1 S proteins mediate cell entry with lower efficiency than the PT, whereas BANAL-20-52 exhibits the highest efficiency (Fig. 1C).

### CX1 and BANAL-20-52 exhibit potentially broader host range than SARS-CoV-2 PT

Since the RBDs of CX1 and BANAL-20-52 resemble that of SARS-CoV-2, it is highly possible that these CoVs also have a broad potential host range. To evaluate such risk, we used flow cytometry and SPR assays to assess the receptor binding spectra among ACE2 orthologs from 21 species including humans, pets (cat [*Felis catus*], dog [*Canis lupus familiaris*]), domestic animals (horse [*Equus caballus*], bovine [*Bos taurus*], goat [*Capra hircus*], sheep [*Ovis aries*], pig [*Sus scrofa*]), wildlife animals (rabbit [*Oryctolagus cuniculus*], mouse [*Mus musculus*], golden hamster [*Mesocricetus auratus*], Chinese hamster [*Cricetulus griseus*], civet [*Paguma larvata*], fox [*Vulpes vulpes*], raccoon dog [*Nyctereutes procyonoides*], wild Bactrian camel [*Camelus ferus*], lesser hedgehog ternec [*Echinops telfairi*]), bats (least horseshoe bat [*Rhinolophus pusillus*], greater horseshoe bat [*Rhinolophus ferrumequinum*], fulvous fruit bat [*Rousettus leschenaultii*]), and marine mammal (minke whale [*Balaenoptera acutorostrata scammoni*]). SARS-CoV-2 PT was also included in the SPR assays for comparison.

**Fig 2:**
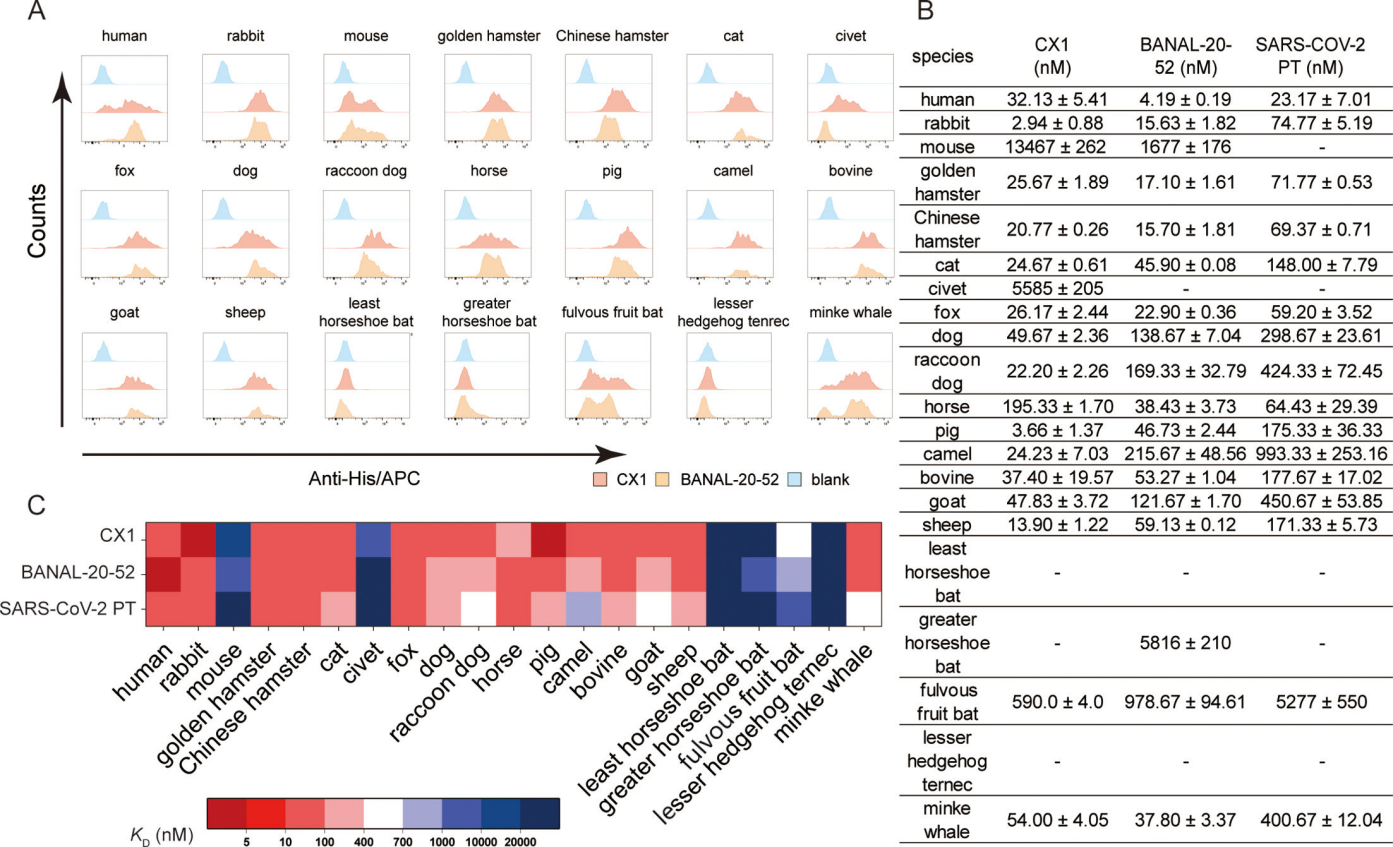
Potential host range of CX1 and BANAL-20-52. (**A**) Flow cytometry analysis of binding between 21 ACE2 orthologs and CX1-RBD and BANAL-20-52-RBD. BHK-21 cells expressing enhanced green fluorescent protein (eGFP)-fused ACE2 orthologs were incubated with the His-tagged CX1-RBD and BANAL-20-52-RBD. Anti-His/allophycocyanin (APC) antibody was used to detect His-tagged proteins. Distribution of APC intensity among eGFP-positive cells is presented as a histogram. The His-tagged SARS-CoV-2 N-terminal domain (NTD) was used as a blank control. (**B**) Binding affinities among ACE2 orthologs to RBDs from CX1 (red), BANAL-20-52-RBD (yellow), and SARS-CoV-2 PT (blue). The *K*_D_ is presented as mean ± SD from three independent experiments. (**C**) Heat map of binding affinities (nM) of CX1 and SARS-CoV-2 PT-RBD with indicated ACE2 orthologs.

Overall, flow cytometry and SPR demonstrated a consistent receptor binding pattern (Fig. 2; Fig. S4). Among the CX1-susceptible species, rabbits and pigs demonstrated the highest affinities with *K*_D_ < 5 nM. Eleven species, namely golden hamster, Chinese hamster, cat, fox, dog, raccoon dog, wild Bactrian camel, bovine, goat, sheep, and minke whale, demonstrated relatively strong affinities, with *K*_D_ of two-digit numbers. Civet exhibited mild binding of 5.5 µM (Fig. 2B and C). Particularly, CX1 demonstrated binding to the mouse, which can be attributed to its N501Y substitution. On the other hand, BANAL-20-52-RBD showed strong binding (*K*_D_ < 100 nM) to 10 species, namely rabbit, golden hamster, Chinese hamster, cat, fox, horse, pig, bovine, sheep, and minke whale. Notably, BANAL-20-52, although lacking Y501, also demonstrated binding to mouse ACE2.

### N370-glycan stabilizes the close conformation for S protein by strengthening inter-protomer interactions

To obtain structural insight into the S proteins of CX1 and BANAL-20-52, we determined the cryo-EM structures of these two S proteins at resolutions of 2.74 Å and 2.50 Å, respectively (Fig. S5 and S6; Table S1). For both S proteins, only a closed conformation was observed, with all three RBDs in the down state (Fig. S7A and B). The BANAL-20-52 S structure is determined with a higher resolution than the previously reported 3.50 Å structure (PDB: 8HXJ) (9). Structural comparison demonstrates resembling architecture between our structure and the published one, with the root mean square deviation (RMSD) of 1.734 Å for 2,850 Cα atoms. Notably, in our BANAL-20-52 S structure, three free linoleic acid (LA) molecules are observed, which are embedded in hydrophobic pockets within the three RBDs (Fig. S7C). Comparison of the hydrophobic pocket surrounding the LA molecules reveals that Y365 in the previous structure blocks the gate of the pocket (Fig. S7D). Further comparison with the LA-present SARS-CoV-2 structure (PDB: 6ZB5) reveals that the LA molecule in BANAL-20-52 S is buried deeper in the pocket, of which the acidic headgroup no longer interacts with the adjacent RBD (Fig. S7E) (28).

**Fig 3:**
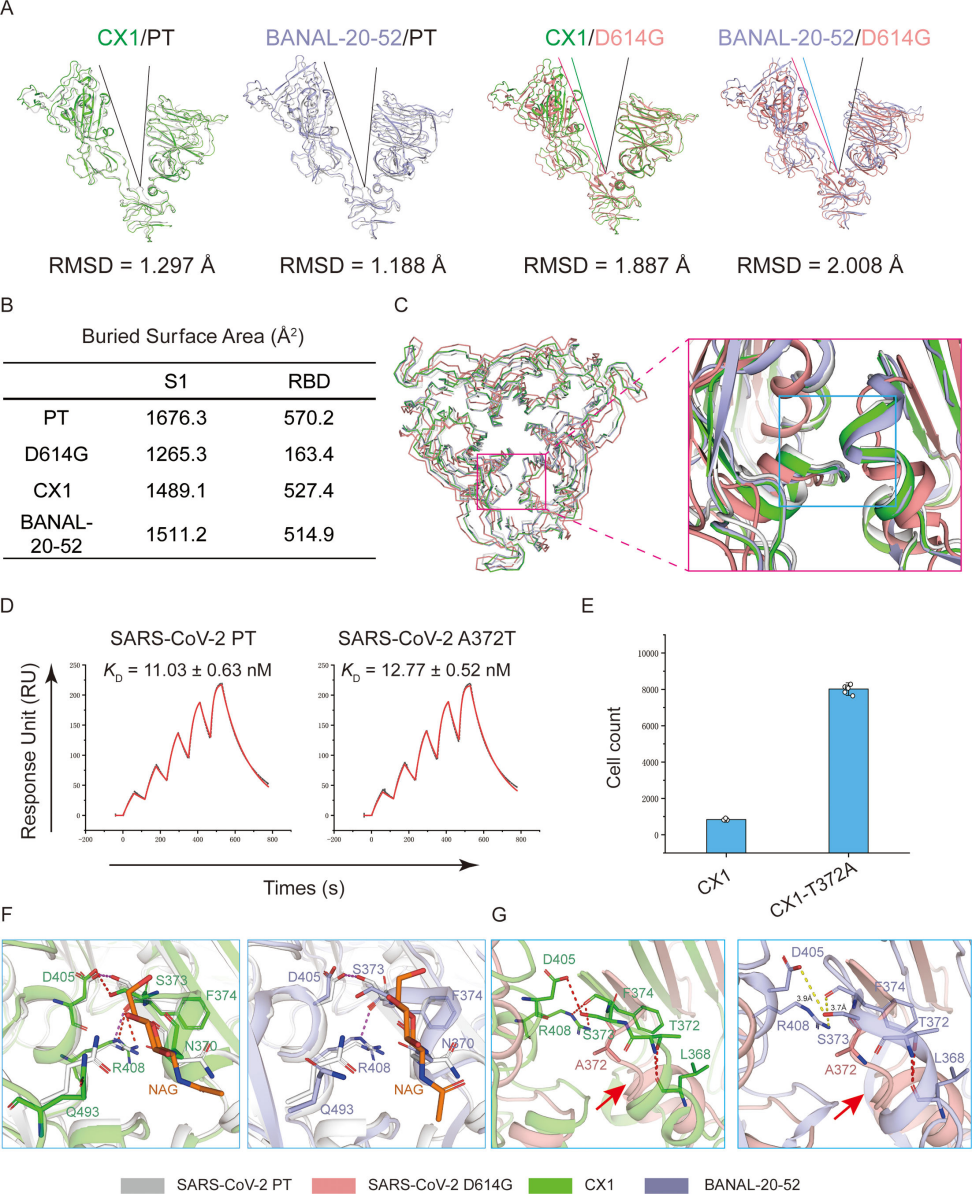
Structural resemblance of CX1 and BANAL-20-52 S with SARS-CoV-2 PT. (**A**) Structural alignment of S1 subunits of SARS-CoV-2 PT (gray), D614G (pink), CX1 (green), and BANAL-20-52 (violet) and comparison of the opening angles between RBD and NTD. (**B**) The BSAs of PT, D614G, CX1, and BANAL-20-52. The numbers indicate the average BSA of three interfaces between adjacent subunits. (**C**) Structural alignment of the RBDs of PT, D614G, CX1, and BANAL-20-52. (**D**) SPR assay of SARS-CoV-2 PT and SARS-CoV-2 A372T-RBDs with hACE2. Black and red lines represent the actual and fitted curves, respectively. The *K*_D_ is presented as mean ± SD from three independent repeats. (**E**) Pseudovirus assay of CX1 and CX1-T372A. The assay was conducted on Vero cells. Data were presented as mean ± SD. Two independent experiments were performed with similar results. (**F**) Structural details of T372A and surrounding residues in SARS-CoV-2 PT (gray), CX1 (green), and BANAL-20-52 (violet). Residues involved in RBD-RBD interaction are presented as sticks. The N370-glycans of CX1 and BANAL-20-52-RBD are colored in orange. Hydrogen bonds formed between adjacent RBDs in PT and CX1/BANAL-20-52 are colored in purple and red, respectively. (**G**) Structural details of T372A and surrounding residues among SARS-CoV-2 D614G (pink), CX1 (green), and BANAL-20-52 (violet). The N370-glycan is hidden for clear visualization of the main chain. The red arrow indicates the loosened ring of α-helix from SARS-CoV-2 D614G. Residues involved in RBD-RBD interaction are presented as sticks. Hydrogen bonds between adjacent CX1-RBDs are presented as red dashes.

We further compared the protomers of the two S proteins with those of the SARS-CoV-2 PT (PDB: 6XR8) and D614G variant (PDB: 7KRQ) (Fig. S8A) (29, 30). The RMSD of CX1 and BANAL-20-52 protomers from the PT are 1.798 Å and 1.692 Å, respectively. It is interesting to note that the RMSD between the D614G and PT is the highest (2.035 Å), demonstrating the extensive structural resemblance of S proteins of CX1 and BANAL-20-52 with that of SARS-CoV-2 PT. More detailed comparison revealed that the deviation between D614G and the two related CoVs is mainly contributed by the S1 subunit, with RMSD of 1.887 Å and 2.008 Å, respectively (Fig. 3A; Fig. S8B). Particularly, the opening angles between N-terminal domain (NTD) and RBD of CX1 and BANAL-20-52 protomers are smaller than that of D614G, indicating that NTD and RBD clamp adjacent RBD more tightly than D614G. On the other hand, no significant difference was observed between PT and the two S proteins (Fig. 3A). We further compared the buried surface area (BSA) between adjacent S1 and RBD. The results showed that the BSA between S1 subunits of the PT and the three related CoVs is comparable (1,489.1–1,676.3 Å^2^), but larger than the D614G (1,265.3 Å^2^). The BSA of the RBDs, on the other hand, further demonstrated a more than threefold difference (514.9–570.2 Å^2^ vs 163.4 Å^2^), further exhibiting a much more compact S1 packing (Fig. 3B). Consistently, superimposition of RBDs demonstrated apparently looser RBD packing of D614G than others (Fig. 3C). The compact packing of S1 subunit suggests the preference of CX1 and BANAL-20-52 S proteins to adopting a closed conformation, consistent with the fact that only a closed conformation is observed in structural determination of the two S proteins (Fig. S5 and S6).

Then, we tried to explore what influences the conformation of CX1 and BANAL-20-52 S proteins. T372A is a substitution exclusive to SARS-CoV-2, which is present in all the SARS-CoV-2 sequences but not in the related animal CoVs, which largely facilitates the virus entry (27, 31). Two mechanisms have been proposed to explain the facilitating role of T372A. Kang et al*.* proposed that this substitution enhanced the binding affinity of RBD with hACE2 (31). Another recent study proposed that T372 forms an N-glycosylation motif with N370, and the N370-glycan prevents RBD erection (9). However, the study compares BANAL-20-52 S protein with Omicron BA.2.75, which contains a large number of mutations compared to the PT. To further elucidate the role of T372A in receptor recognition, we introduced A372T to PT-RBD and measured their binding affinity with hACE2. No significant difference was observed (11.03 nM vs 12.77 nM), indicating that T372A is not directly involved in hACE2/RBD interaction (Fig. 3D). In turn, we introduced T372A to CX1 pseudovirus, which demonstrated a drastic increase in the entry efficiency (Fig. 3E), consistent with a previous report (27).

We further compared the structural details of T372A and its surrounding residues. Comparison of CX1 and BANAL-20-52 to PT demonstrated a largely resembling RBD-RBD interaction, with S373 and F374 forming a hydrogen bond (H-bond) network with D405 and R408 of neighboring RBD, respectively. Notably, N370-glycan forms van der Waals (vdw) interactions with Q493 of the adjacent RBD, thus strengthening the RBD-RBD interaction and stabilizing the down state of RBDs (Fig. 3F). On the other hand, the RBDs of D614G do not form H-bonds between each other, indicating a loosened packing. Additionally, in S proteins of PT and two related CoVs, the residues adjacent to site 372 form a two-turn α-helix (365-373), whereas residues of D614G only form one turn (365-379) (Fig. 3G).

### CX1-RBD and BANAL-20-52-RBD bind to hACE2 in canonical modes

To elucidate the molecular mechanism of CX1-RBD recognizing hACE2, we tried to determine the cryo-EM structures of CX1 S and BANAL-20-52 S complexed with hACE2 (Table S1). Interestingly, when preparing the sample of CX1 S/hACE2 and BANAL-20-52 S/hACE2 complexes, only part of the S1 subunit complexed with hACE2 was observed, indicating an unexpected dissociation (Fig. S9, S10, S11A and B). To verify whether such dissociation occurs upon hACE2 binding, we purified hACE2 as well as CX1 and BANAL-20-52 S trimers, mixed the proteins (molar ratio of S trimer: hACE2 as 1:5, consistent with cryo-EM sample preparation), and performed analytical ultracentrifugation after overnight incubation at 4°C. The molecular weight indicated that one CX1 or BANAL-20-52 S trimer is bound by two hACE2 molecules, and no S1 dissociation is observed, suggesting that the dissociation may have occurred during the cryo-EM sample preparation (Fig. S11C).

**Fig 4:**
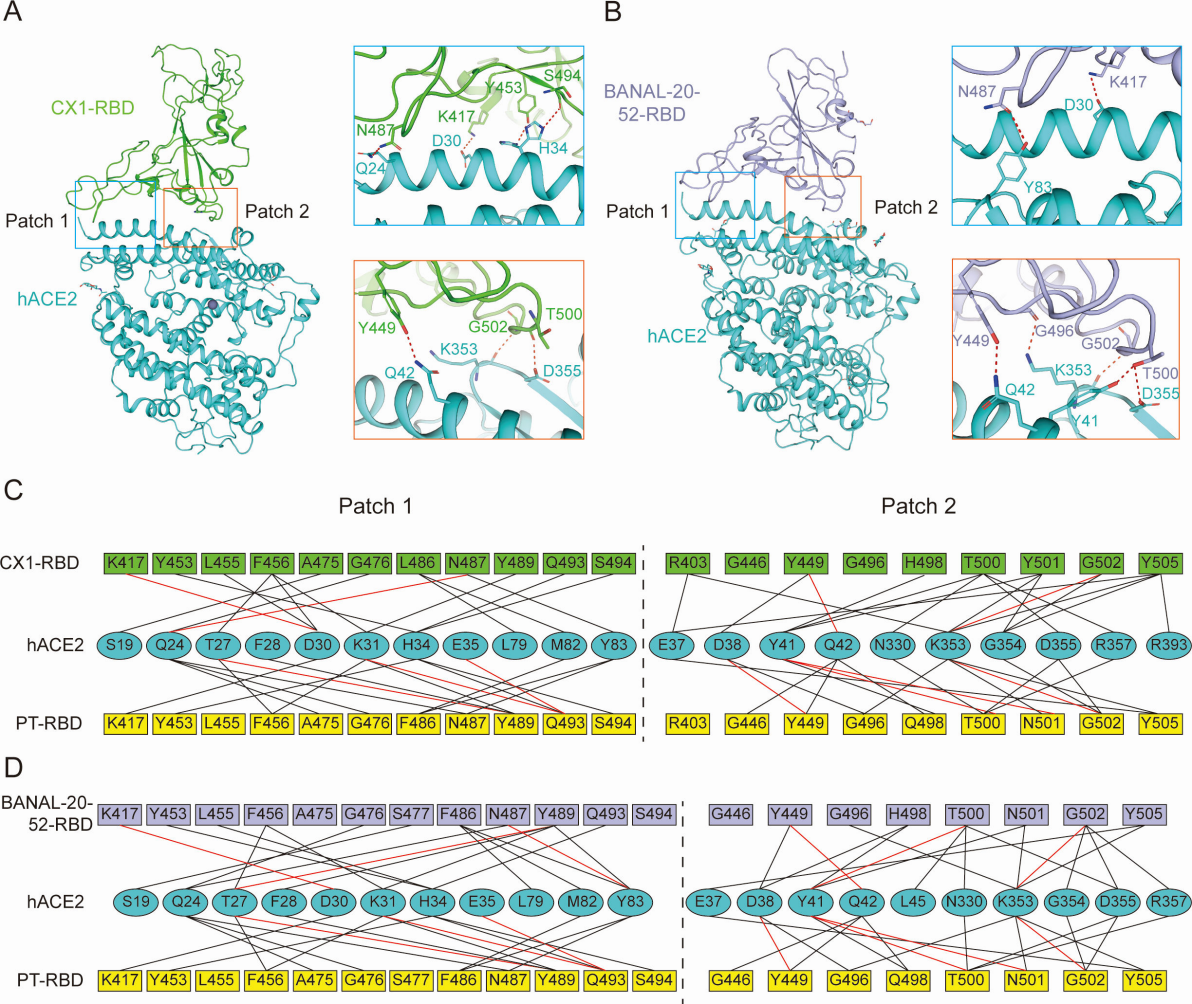
Receptor binding characteristics of CX1-RBD and BANAL-20-52-RBD to hACE2. (**A**) Architecture of CX1-RBD complexed with hACE2 (left) and H-bond network of CX1-RBD complexed with hACE2 (right). Residues involved in H-bond formation are presented as sticks. (**B**) Architecture of BANAL-20-52-RBD complexed with hACE2 (left) and H-bond network of BANAL-20-52-RBD complexed with hACE2 (right). Residues involved in H-bond interaction are presented as sticks. (**C, D**) Interaction network of hACE2 binding to CX1 (**C**) and BANAL-20-52-RBD (**D**) and comparison with SARS-CoV-2 PT-RBD. Residues interacting with vdw contacts and polar interactions (H-bond and salt bridge) are connected by black and red lines, respectively.

The binding interface between CX1-RBD and hACE2 was clearly observed and demonstrated a similar pattern to SARS-CoV-2-RBD (Fig. 4A; Fig. S11A). In Patch 1, N487 of CX1-RBD forms an H-bond with Q24 of hACE2, whereas K417 of CX1-RBD forms a salt bridge with D30 of hACE2. Two conformations of hACE2 H34 were observed, forming an H-bond with Y453 and S494 of CX1-RBD, respectively. In Patch 2, Y449, T500, and G502 of CX1-RBD form an H-bond with Q42, D355, and K353 of hACE2, respectively (Fig. 4A). On the other hand, in Patch 1 of BANAL-20-52-RBD/hACE2 interface, the salt bridge between K417 and D30 is conserved, whereas N487 forms an H-bond with Y83. In Patch 2, Y449, G496, T500, and G502 of the RBD form an H-bond network with Y41, Q42, K353, and D355 (Fig. 4B; Fig. S11B).

We further analyzed the binding interface of CX1-RBD and BANAL-20-52-RBD with hACE2 and compared it with SARS-CoV-2 PT (PDB: 6M17) (32). Among the RBD-interacting residues, S19 and L79 interact with the two related CoV RBDs but not the PT-RBD, while F28 binds to the BANAL-20-52 and PT-RBD but not the CX1-RBD. L45 and E35 bind exclusively to BANAL-20-52 and PT-RBD, respectively. As for the ACE2-interacting residues of RBDs, R403 of CX1, S477 of BANAL-20-52, and G446 of PT bind to hACE2 while their counterparts of the other two RBDs do not (Fig. 4C and D; Fig. S11D).

### Y501 shows a synergetic effect with R498 but is inter-replaceable with H498 for hACE2 binding

**Fig 5:**
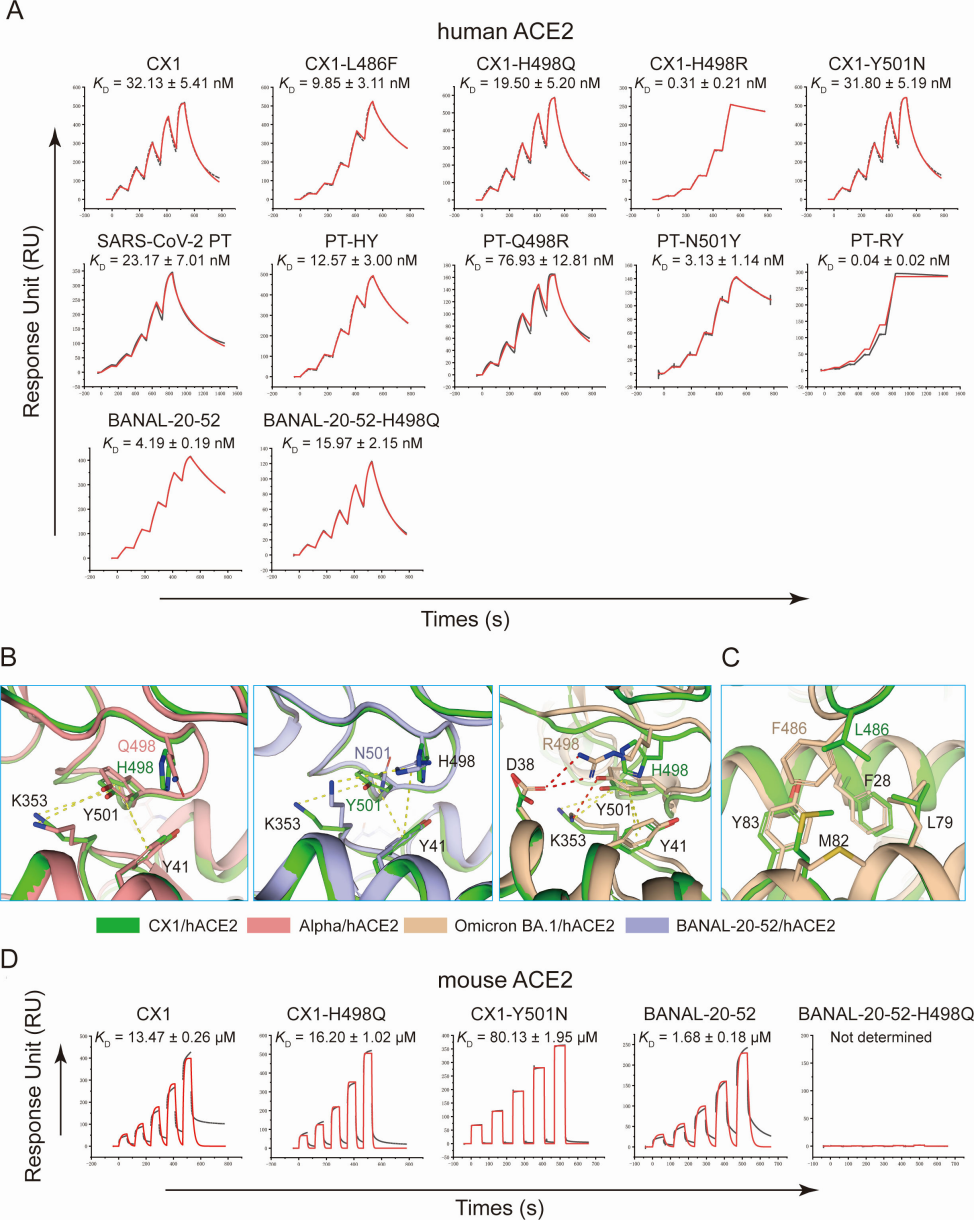
Key residues affecting receptor binding of CX1-RBD and BANAL-20-52-RBD. (**A**) SPR assays of PT-RBD, CX1-RBD, BANAL-20-52-RBD, and their mutants with hACE2. Black and red lines represent the actual and fitted curves, respectively. (**B, C**) Structural details of site 498/501 (**B**) and 486 (**C**). Involved residues are presented as sticks. Complexes of hACE2 with CX1-RBD, SARS-CoV-2 Alpha-RBD, Omicron BA.1-RBD, and BANAL-20-52-RBD are colored in green, salmon, wheat, and violet, respectively. H-bonds are presented as red dashes. π-π and π-cation interactions are presented as yellow dashes. (**D**) SPR assay of mouse ACE2 binding with wild-type CX1-RBD and BANAL-20-52-RBD and their mutants. The *K*_D_ is presented as mean ± SD from three independent repeats.

Compared with PT-RBD, CX1-RBD contains three distinct sites on its ACE2-interacting residues, namely F486L, Q498H, and N501Y, and BANAL-20-52 carries only Q498H. On the other hand, Q498R is another common substitution and is fixed in all the Omicron sub-variants, suggesting its important role in receptor binding. To evaluate their effects on ACE2 recognition, we constructed mutants of CX1-RBD containing individually L486F, H498Q, H498R, and Y501N and BANAL-20-52 mutants containing H498Q. Additionally, we introduced both Q498H and N501Y to PT-RBD and constructed PT-HY carrying both Q498H and N501Y. The SPR assay with hACE2 demonstrates that CX1-H498Q brought no significant difference to RBD binding, but BANAL-20-52-H498Q showed a 3.81-fold decreased affinity, indicating that the effect of H498Q is influenced by other factors. On the other hand, CX1-Y501N showed no decrease in binding affinity, which is interesting as Y501 is recognized as a preferred residue in hACE2 binding (22, 33). Meanwhile, L486F and H498R enhanced binding affinities by 3.3 and drastic 104 folds, respectively. As previous studies reported that Q498R alone has no enhancing effect on binding affinity (34, 35), such a drastic increase indicats a strong Q498R-N501Y synergetic effect. PT-HY demonstrated similar affinity with CX1-L486F (12.57 nM vs 9.85 nM), consistent with the fact that they share identical residues on the binding interface (Fig. 5A).

To elucidate the molecular mechanism of these substitutions impacting receptor recognition, we compared the structural details of hACE2 binding with RBDs of CX1, BANAL-20-52, PT, Alpha (N501Y), and Omicron BA.1. It is reported that N501Y facilitates hACE2 binding by forming π-π interaction with Y41 and π-cation interaction with K353 of hACE2 (22). The imidazole ring of H498 can also participate in such π-π and π-cation interactions. Thus, when the RBD carries H498 and Y501 at the same time, these interactions are saturated and can only be formed with either H498 or Y501, but not with both simultaneously. In BANAL-20-52-RBD/hACE2, when Y501 was substituted by N501, although Y41 and K353 can no longer form π-π or π-cation interactions with Y501, the orientation of H498 is altered and replaces the π-π and π-cation interactions, and the binding affinity is rescued. Thus, the CX1 (H498/Y501), CX1-H498Q (Q498/Y501), and CX1-Y501N (H498/N501) demonstrated comparable affinities with hACE2. Besides, such replaceability can also explain why CX1-H498Q (Q498/Y501) demonstrated similar affinity to CX1 (H498/Y501), but BANAL-20-52-H498Q (Q498/N501) showed 3.8-fold lower than wild type (H498/N501). As for the synergetic effect of Q498R and N501Y, Q498R forms an H-bond and a salt bridge with D38, largely strengthening the ACE2 interaction (Fig. 5B). The F486 forms a hydrophobic patch with F28, L79, M82, and Y83 of hACE2, whereas the shorter side chain of L486 only interacts with M82 and L79 (Fig. 5C).

To validate the synergetic effect of Q498R and N501Y, we further constructed mutants of SARS-CoV-2 PT, namely PT-Q498R, PT-N501Y (RBD of SARS-CoV-2 Alpha variant), and PT-RY (Q498R and N501Y introduced) and measured their affinities binding to hACE2. PT-RY exhibited a 579-fold increase in binding affinity, while N501Y resulted in strengthened binding but to a much lesser extent (7.4-fold), and Q498R decreased hACE2 binding affinity (Fig. 5A). Such results are consistent with our explanation and further validate the synergetic effect.

Then, we further explored whether the inter-replaceability between Q498H and N501Y applies for mouse ACE2 binding. We measured the binding affinities of CX1-H498Q, CX1-Y501N, and BANAL-20-52-H498Q with mouse ACE2. The results showed that the two CX1 mutants demonstrated binding with mouse ACE2, while H498Q fully abrogated mouse ACE2 binding of BANAL-20-52-RBD (Fig. 5D).

We further investigated whether such interplay can be applied to pseudovirus entry efficiency. The point mutations of SARS-CoV-2 PT and CX1 have no significant effect on S protein incorporation, whereas H498Q largely enhanced the S protein incorporation of BANAL-20-52 (Fig. S12A). Although R498/Y501 largely increased hACE2 binding, no enhancement was observed in entry efficiency. On the other hand, pseudovirions containing H498 and/or Y501 showed efficient entry (Fig. S12B). Only mild infections are observed on cells expressing mouse ACE2 (Fig. S12B).

## DISCUSSION

Analyses on RBD/hACE2 complexes and the receptor binding spectra of CX1 and BANAL-20-52 support that some animal CoVs in fact pose a risk of interspecies transmission. Additionally, the pseudovirus assays in this study and other reports also indicate that S proteins of the related CoVs can obtain a significantly enhanced ability to mediate cell entry by simply acquiring T372A single substitution or an FCS (9, 27, 36). Therefore, surveillance on zoonotic CoVs to forewarn such recombination is urgently needed.

CX1-RBD and BANAL-20-52-RBD demonstrate broader receptor binding spectra than SARS-CoV-2 PT. On the other hand, the narrower receptor binding spectrum may also indicate adaptation to humans. Such adaptation has also been observed in the subsequential evolution of SARS-CoV-2 variants/sub-variants, where N501Y and K417N mutations of SARS-CoV-2 variants decreased binding to ACE2 of horse and raccoon dog, respectively (37, 38). These results also remind us to focus not only on the related CoVs with broad potential hosts but also on CoVs with a narrower host range but higher hACE2 usage.

It is noteworthy that receptor recognition is only one of the multiple steps of the complicated infection process, and infections are affected by many other factors, such as S protein conformation, fusogenicity, protease cleavage, host immune response, *etc*. (39–41). Moreover, strong receptor binding does not necessarily mean high transmissibility, which is reflected partly by the comparable pseudovirus entry of SARS-CoV-2 PT and PT-RY despite the largely increased binding affinity. Such discrepancy has also been discussed in our previous report, in which we found that the binding affinities of dominantly circulating SARS-CoV-2 variants fall within an optimal range (42). On the other hand, the pseudovirus infection assay may also be influenced by underlying factors, such as S protein incorporation (as reflected in Fig. S12), cell sorting, etc. Therefore, results from receptor binding and pseudovirus infection assays require integrated analysis with other data sets to derive robust conclusions. However, receptor binding is an important step in the infection process, and evaluation of the receptor binding may provide guidance for animal surveillance. For example, studies in 2020 reported the binding of ACE2 orthologs from goat, sheep, gorilla, lynx, and puma, and natural infections in these species were later observed in field surveys (23, 24, 26).

In this study, we have elucidated the interplay between Y501 and H498/R498. Q498H can replace N501Y in not only hACE2 binding but also mouse ACE2 binding. These findings fill in another piece for the jigsaw puzzle of CoV interspecies transmission. As CX1-RBD contains both Q498H and N501Y substitutions, these results further address the importance of CX1 and necessitate the inclusion of Q498H as an important surveillance indicator. On the other hand, Y501 and R498/Y501 have been reported as epistatic to other immune-evasive mutations in Omicron sub-variants (34, 35, 43). In this study, we elucidated how R498/Y501 exerts such a strong synergetic effect to enhance hACE2 binding, which may serve as a “ballast” to maintain receptor recognition and allow subsequent mutations.

In summary, evaluation of the receptor binding spectra of CX1 and BANAL-20-52 unveils a potential broader host range and higher affinities to most tested species than that of SARS-CoV-2 PT. Determination and analysis of cryo-EM structures of CX1-RBD and BANAL-20-52-RBD complexed with hACE2 revealed Q498H/N501Y inter-replaceability and R498/Y501 synergetic effect. These results provide the basis for interspecies transmission and address the importance of surveillance on potential emerging CoVs.

## MATERIALS AND METHODS

### Plasmids

The coding sequences of the CX1-RBD (residues 319-541, https://doi.org/10.5281/zenodo.7941061), SARS-COV-2 PT-RBD (GISAID: EPI_ISL_402119), BANAL-20-52-RBD (GISAID: EPI_ISL_4302644), mutated CX1-RBD (L486F, H498Q, H498R, Y501N), mutated SARS-COV-2 PT-RBDs (A372T, HY, Q498R, N501Y, RY), and the peptidase domain (PD) of hACE2 (residues 19-615, UniProt: Q9BYF1) were cloned into the pCAGGS vector with a C-terminal 6× HisTag. The ectodomains of 23 ACE2s fused with the Fc domain of mouse IgG (mFc) were individually cloned into the pCAGGS vector for SPR. The full-length ACE2 coding sequences of 21 species were synthesized and, respectively, cloned into the pEGFPN1 vector used for flow cytometry. The ectodomains of S proteins of CX1 and BANAL-20-52 with stabilizing mutations (CX1: F813P, A888P, A895P, A938P, K982P, and V983P; BANAL-20-52: F813P, A888P, A895P, A938P, K982P, and V983P, based on SARS-CoV-2 residue numbering) are fused with C-terminal T4 fibritin trimerization domain and a Strep-Tag-II and cloned into pcDNA3.1.

### Protein expression and purification

The CX1-RBD, BANAL-20-52-RBD, SARS-COV-2 PT-RBD, mutated CX1-RBD, mutated SARS-COV-2 PT-RBDs, and hACE2 cloned in pCAGGS were expressed in Expi293F cells cultured in 293F Hi-exp CDM (cat. no. AC601501, OPM_Biosciences). The plasmids were transiently transfected into cells and cultivated for 5 days. Cell culture supernatants were collected, filtered through 0.22 µm filters, and purified by His-Trap HP (GE Healthcare) and Superdex 200 Increase 10/300 GL (GE Healthcare) chromatography. Purified proteins were stored in PBS (1.8 mM KH_2_PO_4_, 10 mM Na_2_HPO_4_ [pH 7.4], 137 mM NaCl, 2.7 mM KCl). The CX1 S and BANAL-20-52 S were transfected into Expi293F cells and cultivated for 3 days. The cell culture supernatants were purified by StrepTrap HP (GE Healthcare) and HiLoad 16/600 Superose 6 pg column.

### Flow cytometry

The ACE2-pEGFP-N1 plasmids were transfected into BHK-21 cells. When cell fluorescence could be observed with a fluorescence microscope within 48 h, the cells were collected, washed twice with PBS, and distributed into 96-well plates. The cells were incubated with test proteins (CX1-RBD, BANAL-20-52-RBD, and SARS-CoV-2-NTD with 6× HisTag) at a concentration of 30 µg/mL at 4°C for 30 min. Then, the cells were washed three times with PBS and stained with anti-His/allophycocyanin antibody (1:500, Miltenyi Biotec) at 4°C for 30 min. Finally, the cells were analyzed using a BD FACSCanto (BD Biosciences) after washing thrice with PBS. All data were analyzed using FlowJo V10.

### SPR

The binding affinities between the mFc-tagged ACE2s and CX1-RBD, BANAL-20-52-RBD, and SARS-CoV-2 PT-RBD were measured using a BIAcore 8K (GE Healthcare) carried out at 25°C in single-cycle mode. The PBST (PBS + 0.005% [vol/vol] Tween 20) was used as the running buffer. First, mFc-tagged ACE2s were immobilized on the CM5 biosensor chip (GE Healthcare) using an amine-coupling chemistry protocol (GE Healthcare). Then, the CX1-RBD protein was serially diluted and flowed through the chip surface, and the binding response was measured. The equilibrium dissociation constants (*K*_D_) of each pair of binding were calculated using BIAcore 8K Evaluation Software (GE Healthcare) by fitting to a 1:1 Langmuir binding model. The SPR assay of hACE2 binding with CX1-RBD, SARS-CoV-2 PT-RBD, and CX1 mutants follows the same protocol with ACE2 immobilized and RBDs as flow-through.

### Pseudovirus entry assay

Non-replicable VSV-ΔG-GFP-based PT, CX1, BANAL-20-52, and CX1-T372A pseudoviruses were prepared as previously described (44). For the entry assay, different pseudoviruses were normalized by dilution according to the qPCR results. The supernatant of 96-well Vero cells was discarded, and 100 µL/well pseudovirus diluent was added to each well. After culturing for 18 h, the fluorescence was counted using a CQ1 confocal image cytometer (Yokogawa). The bar graph was generated using Graphpad Prism 9.2 (https://www.graphpad.com/). Each pseudovirus type was replicated in six wells of Vero cells. Two independent experiments are performed for the pseudovirus assay.

To analyze the level of S proteins in pseudovirions, the pseudovirus-containing supernatant was mixed with 20% (vol/vol) of 5× loading buffer and boiled for 10 min. The samples were then separated on an SDS-PAGE and transferred to a nitrocellulose blot. The S proteins were detected with rabbit polyclonal anti-S2 antibody (1:1,000), and the VSV-M was detected with mouse anti-VSV-M monoclonal antibody (1:1,000, Kf-Ab01404). The blots were further stained with horseradish peroxidase-conjugated goat anti-rabbit IgG or goat anti-mouse IgG and then visualized with Clarity Western ECL substrate (Bio-Rad, Hercules, CA, USA).

For assay in Fig. S12, BHK-21 cells are transfected with enhanced green fluorescent protein (eGFP)-fused human or mouse ACE2. Cells expressing ACE2 are sorted 24 h post-transfection with FACSAria Fusion SORP. The sorted cells are infected after 24 h with the same methodology on Vero cells.

### Cryo-EM sample preparation and data acquisition

To prepare the cryo-EM samples, the CX1 S and BANAL-20-52 S samples were both vitrified using a Vitrobot Mark IV (ThermoFisher Scientific) plunge-freezing device. The BANAL-20-52 S complex sample (4.0 µL, 1.0 mg/mL) was applied to an Au Quantifoil 1.2/1.3 holey carbon grid that was glow discharged for 25 s. The CX1 S complex sample (4.0 µL, 1.47 mg/mL) was applied to a 20 s glow discharged Cu Quantifoil 1.2/1.3 holey carbon grid. The grids above were then blotted using different conditions (blot time 7 s and blot force −10 for BANAL-20-52 S; blot time 4 s and blot force −4 for the CX1 S) at a temperature of 4°C and a humidity level of >98% and plunge frozen into liquid ethane. The CX1-S/hACE2 and BANAL-20-52 S/hACE2 are prepared in a similar process, with a molar ratio of S trimer: hACE2 as 1:5, incubated overnight, and applied to the preparing workflow.

The prepared grids were transferred to a 105 kV Titan Krios transmission electron microscope equipped with a Gatan K3 detector and GIF Quantum energy filter. Movies were collected at 105,000× magnification with a calibrated pixel size of 0.69 Å, over a defocus range of −1.0 μm to −2.0 µm, in super-resolution counting mode with a total dose of 60 e^−^/Å^2^ using EPU (ThermoFisher Scientific) automated acquisition software.

The detailed data processing workflow is summarized in Fig. S4, S5, S8, and S9. All of the raw dose-fractionated image stacks were 2× binned, aligned, dose-weighted, and summed using MotionCor2 (45). The contrast transfer function (CTF) estimation, particle picking and extraction, 2D classification, *ab initio* model generation, and 3D refinements were performed in cryoSPARC v.3.3.1 (46).

For the CX1 S data set, the initial particles were automatically picked and extracted. After extensive 2D classification, good particles were selected to generate the initial models and 3D classification, resulting in three distinct volumes. A dominant class containing 64.06% of the total particles was identified, which displayed clear features of secondary structural elements, especially in the area of the binding interface of the RBD and ACE2. These particles were subjected to homogeneous, non-uniform, and CTF refinements in cryoSPARC v.3.3.1, which yielded a final density map at 2.74 Å resolution estimated by the gold-standard Fourier shell correlation cut-off value of 0.143.

The BANAL-20-52 S data set was processed similarly. A dominant class from five distinct volumes containing 70.60% of the total particles was identified, which displayed clear features of secondary structural elements, especially in the area of the binding interface of the RBD and ACE2. These particles were subjected to homogeneous, non-uniform, and CTF refinements in cryoSPARC v.3.3.1, which yielded a final density map at 2.50 Å resolution.

The CX1-RBD/hACE2 data set was processed similarly. A dominant class from three distinct volumes containing 51.56% of the total particles was identified, which displayed clear features of secondary structural elements, especially in the area of the binding interface of the RBD and ACE2. These particles were subjected to homogeneous, non-uniform, and CTF refinements in cryoSPARC v.3.3.1, which yielded a final density map at 3.04 Å resolution.

The BANAL-20-52-RBD/hACE2 data set was processed similarly. A dominant class from three distinct volumes containing 41.25% of the total particles was identified, which displayed clear features of secondary structural elements, especially in the area of the binding interface of the RBD and ACE2. These particles were subjected to homogeneous, non-uniform, and CTF refinements in cryoSPARC v.3.3.1, which yielded a final density map at 2.95 Å resolution.

### Model building and structure refinement

For the initial model building of the CX1-RBD/hACE2 and BANAL-20-52-RBD/hACE2 complexes, the SARS-CoV-2-S trimer with the PD of hACE2 (PDB: 6LGZ) was used as the starting model and fitted into the corresponding overall cryo-EM maps using UCSF Chimera v.1.15 (47). Mutation and manual adjustment were performed with Coot v.0.9.3 (48). Glycans were added at N-linked glycosylation sites in Coot. Each residue was manually checked with the chemical properties taken into consideration during model building. Several rounds of real-space refinement in Phenix-1.20.1 (49) and manual building in Coot were performed until the final reliable models were obtained. Molprobity (50) was used to validate geometry and check structure quality. Statistics associated with data collection, 3D reconstruction, and model building are summarized in Table S1. Figures were generated using Chimera and PyMol v.2.0 (http://www.pymol.org). The CX1 S and BANAL-20-52 S follow the same procedure with the SARS-CoV-2 S trimer (PDB: 6ZGI) as the starting model.

### Quantification and statistical analysis

#### Binding affinity analysis

*K*_D_ values of SPR experiments were obtained with BIAcore 8K Evaluation Software (GE Healthcare), using a 1:1 binding model. The values indicate the mean ± standard deviation of three independent experiments.

## Data Availability

The cryo-EM density maps and atomic coordinates of CX1 S, BANAL-20-52 S, CX1-RBD/hACE2, and BANAL-20-52-RBD/hACE2 have been deposited to the Electron Microscopy Data Bank (EMDB) and the Protein Data Bank (PDB) with accession numbers EMD-38778, EMD-38773, EMD-38779, and EMD-39695 and 8XYM, 8XYH, 8XYO, and 8YZJ, respectively. Any additional information required to reanalyze the data reported in this work is available from the lead contact upon request.

## SUPPLEMENTAL MATERIAL

Supplemental material jvi.00283-25-s0001.pdf Figures S1 to S12; Table S1.

ASM does not own the copyrights to Supplemental Material that may be linked to, or accessed through, an article. The authors have granted ASM a non-exclusive, world-wide license to publish the Supplemental Material files. Please contact the corresponding author directly for reuse.
