# Supplementary material for: CX1/BtSY2 and BANAL-20-52 exhibit broader receptor binding and higher affinities to multiple animal ACE2 orthologs than SARS-CoV-2 prototype
Source: J Virol. 2025 Jul 10;99(8):e00283-25. doi: 10.1128/jvi.00283-25 (PMC12363175; doi:10.1128/jvi.00283-25)
Supplement: Supplemental material — Figures S1 to S12; Table S1. [file jvi.00283-25-s0001.pdf]

Supporting Information

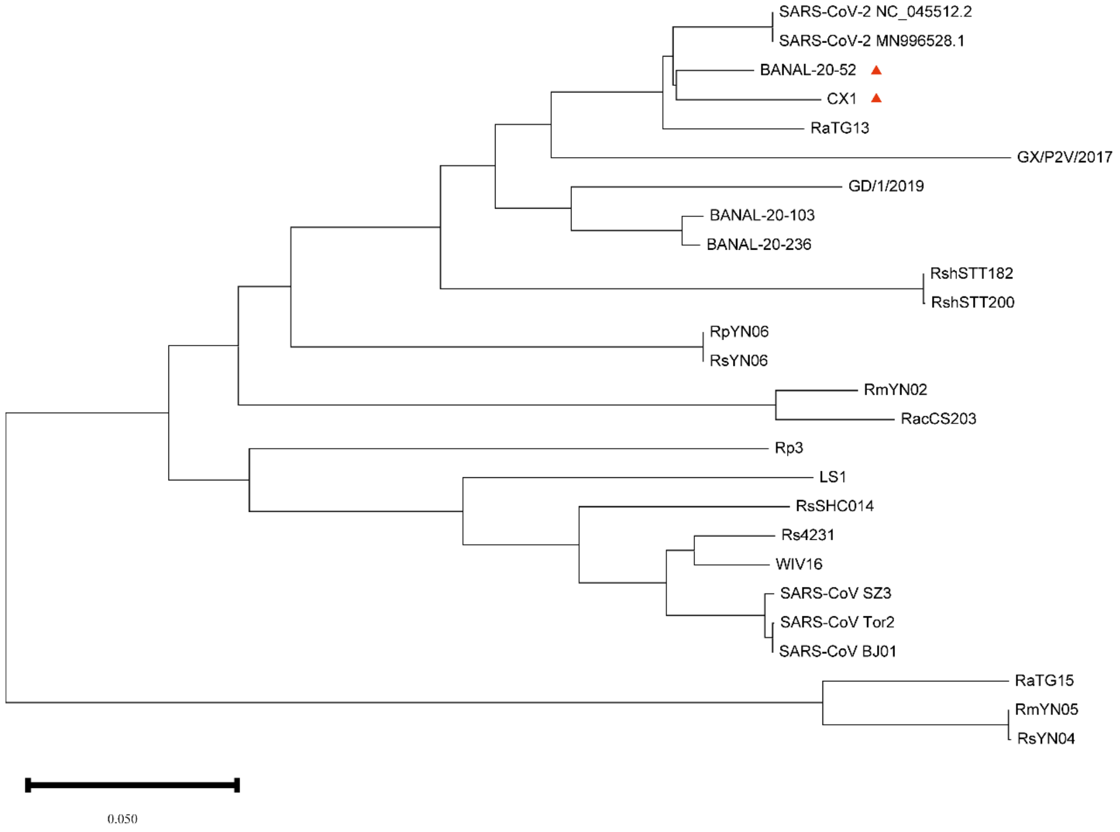

Fig. S1 Phylogenetic tree of SARS-CoV-2, CX1, BANAL-20-52 and representative related CoVs using amino acid sequences of S proteins. CX1 and BANAL-20-52 are labeled with red triangles.

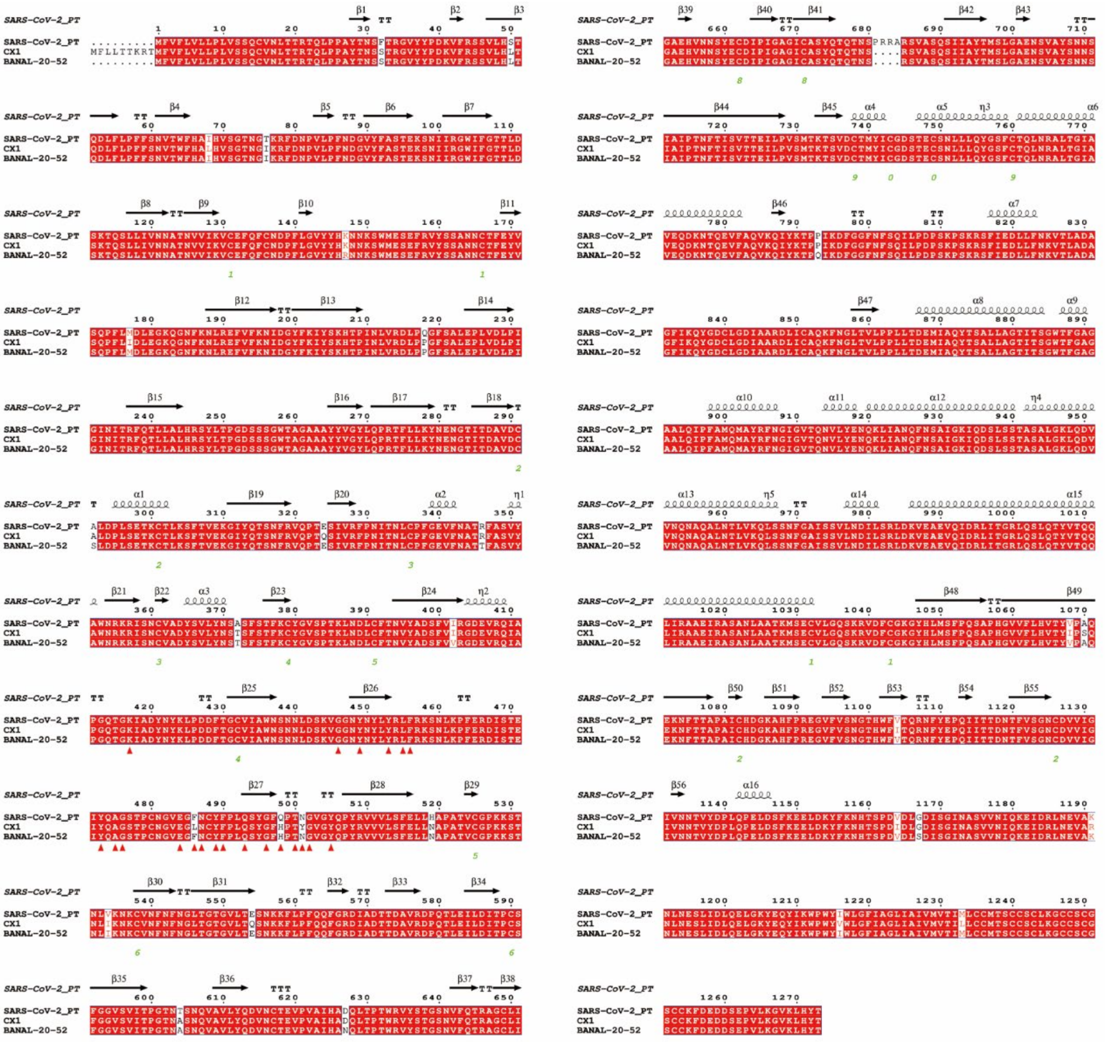

Fig. S2 Sequence alignment of S proteins from SARS-CoV-2 PT, CX1 and BANAL-20-52. Residues involved in the interaction between SARS-CoV-2 PT and hACE2 are labeled with red triangles. Identical residues are highlighted in white on a red background, and residues in red on a white background indicate a similarity score >0.7, considering physio-chemical properties. The alignment was performed by T-COFFEE and visualized by ESPript 3.0.

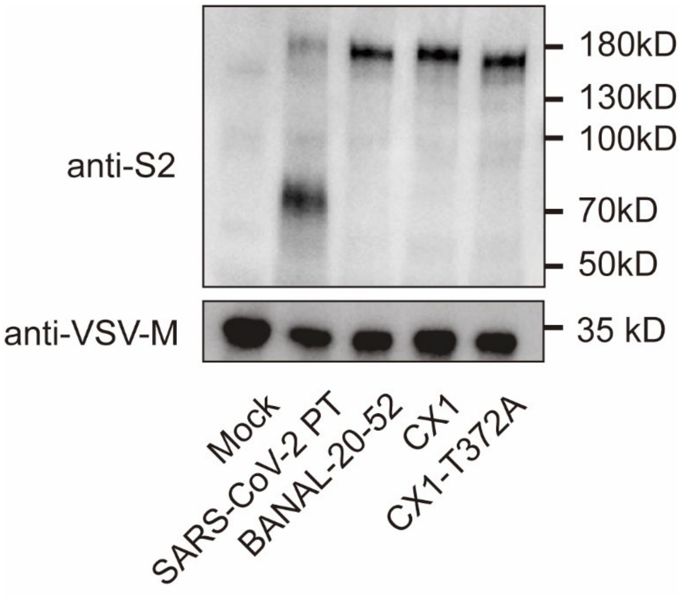

Fig. S3 Detection of S proteins of SARS-CoV-2, BANAL-20-52, CX1 and CX1-T372A in pseudovirions by western blot assay. The S proteins are detected by anti-S2 polyclonal antibodies. VSV-M served as loading controls.

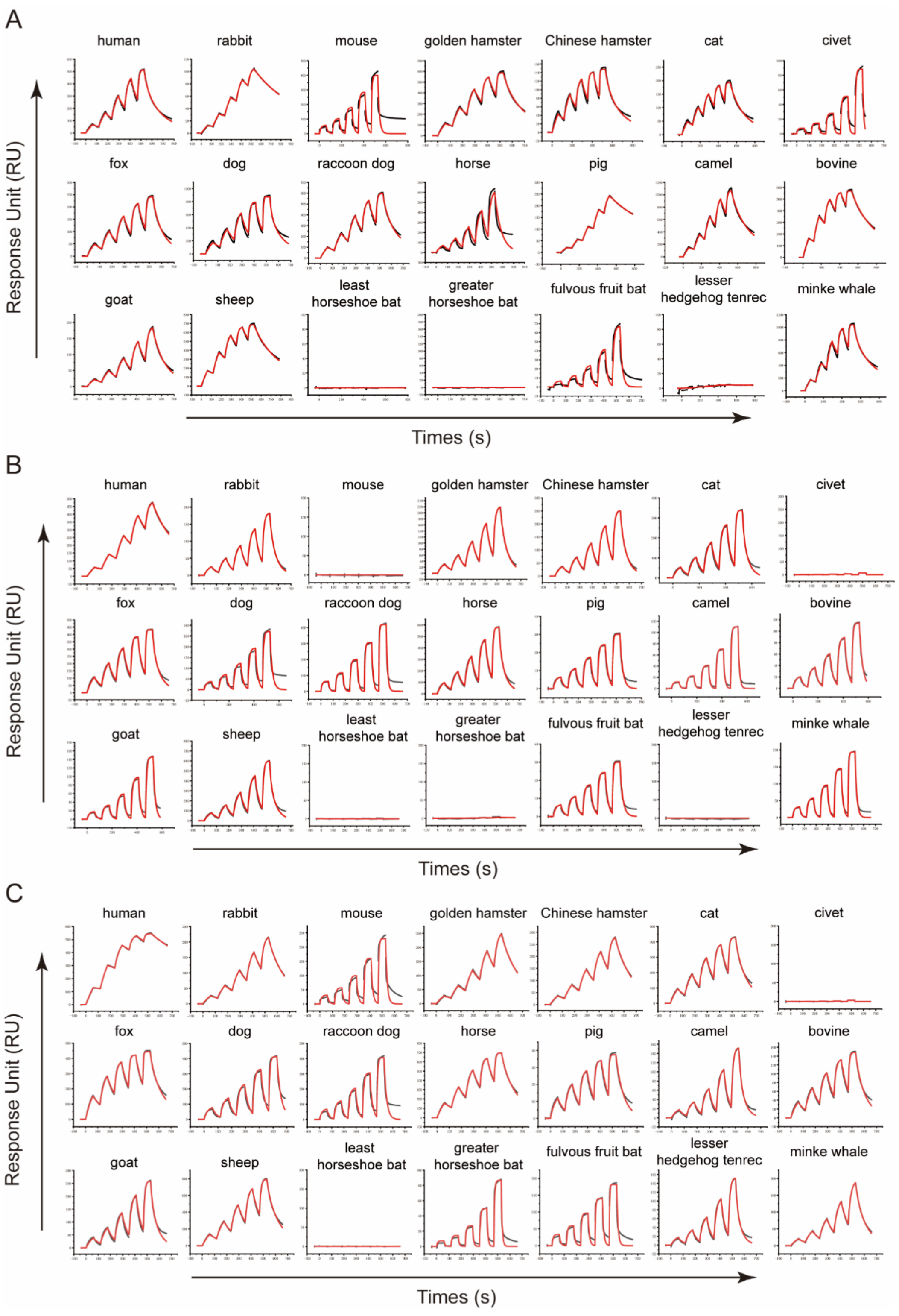

Fig. S4 SPR assay of CX1, BANAL-20-52 and SARS-CoV-2 PT-RBD binding to ACE2 orthologs. The raw and actual curves of CX1 (A), BANAL-20-52 (B) and SARS-CoV-2 PT (C) are presented as black and red curves.

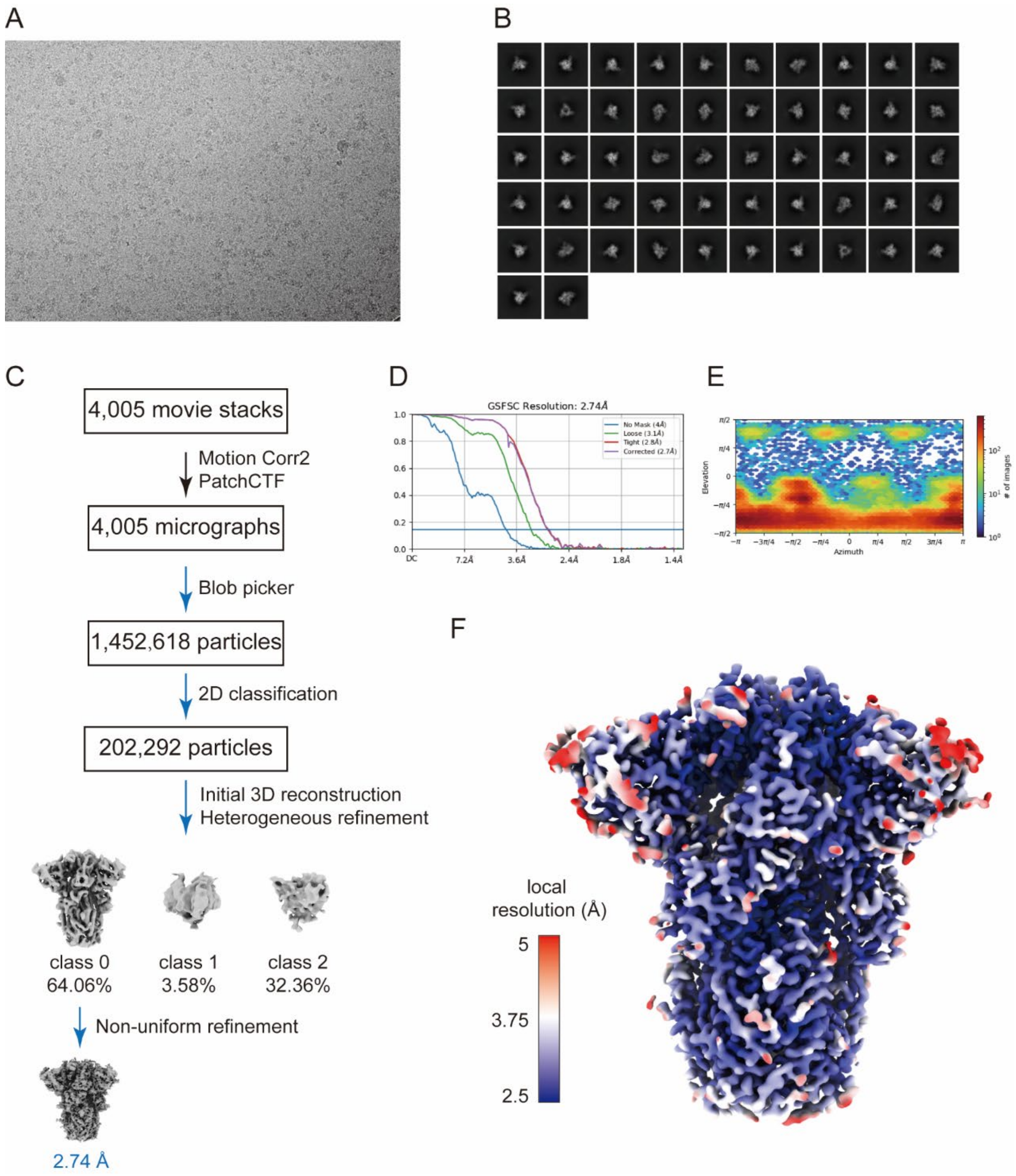

Fig. S5 Cryo-EM data processing of the CX1 S protein. (**A**) A representative electron micrograph. (**B**) 2D classes selected for reconstruction. (**C**) Main steps of image processing. (**D**) Local resolution estimation of the final volume. (**E**) Angular distribution of the particles. (**F**) Resolution distribution of the cryo-EM map, where blue represents high resolution areas, and red represents low resolution areas.

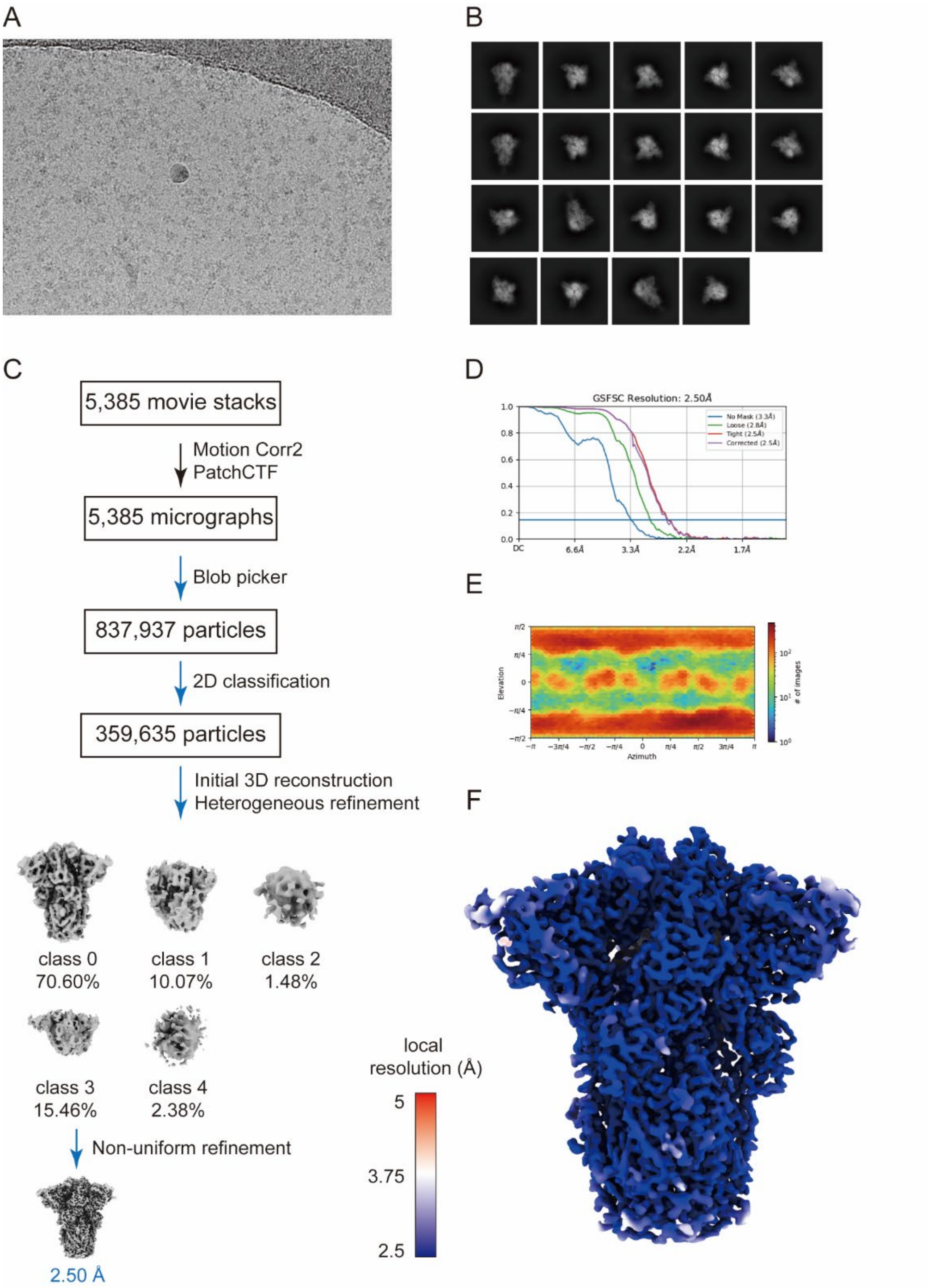

Fig. S6 Cryo-EM data processing of the BANAL-20-52 S protein. (**A**) A representative electron micrograph. (**B**) 2D classes selected for reconstruction. (**C**) Main steps of image processing. (**D**) Local resolution estimation of the final volume. (**E**) Angular distribution of the particles. (**F**) Resolution distribution of the cryo-EM map, where blue represents high resolution areas, and red represents low resolution areas.

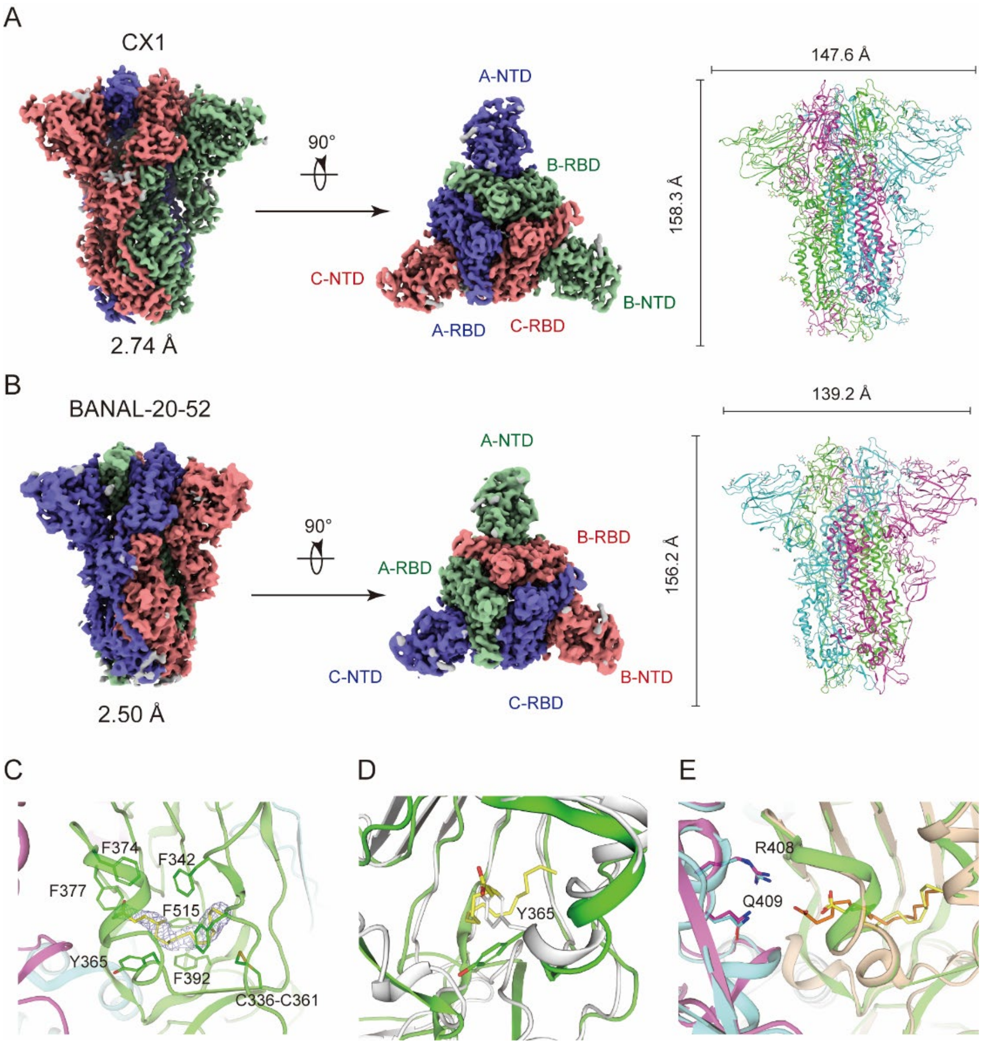

Fig. S7 Overall architectures of CX1 and BANAL-20-52 S proteins and linoleic acid in BANAL-20-52 S protein. (**A, B**) The architecture of S proteins of CX1 (A) and BANAL-20-52 (B). Three protomers are colored in red, green and purple, respectively. (**C**) Cryo-EM map of Linoleic acid (LA) molecule. The hydrophobic residues interacting with LA are presented as sticks. (**D**) Alignment of LA (yellow) and surrounding residues of BANAL-20-52 S protein structure in this study (magenta) and previous study by Ou *et al*. (white) (**E**) Alignment of LA and surrounding residues of BANAL-20-52 and SARS-CoV-2 PT S proteins. RBDs and LA of BANAL-20-52 are colored in green, magenta and yellow, while SARS-CoV-2 in wheat, cyan and orange. R408 and Q409, which interacts with LA of SARS-CoV-2, are presented as sticks.

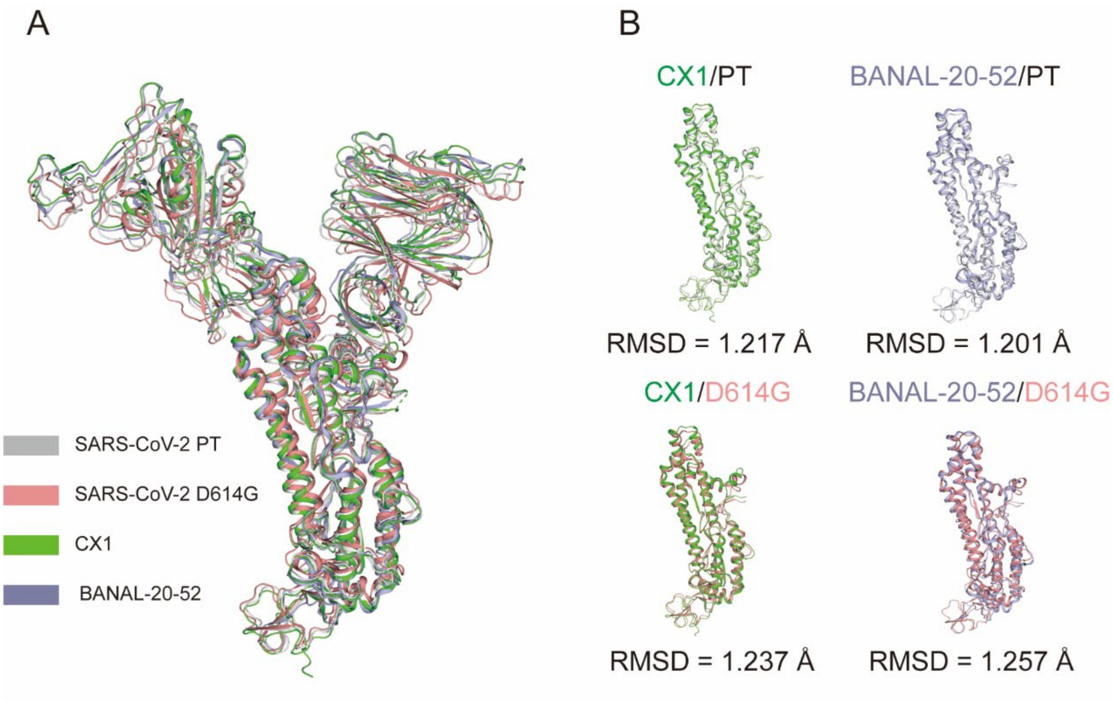

Fig. S8 Alignment of CX1 and BANAL-20-52 S proteins with SARS-CoV-2. (**A**) Alignment of S protomers of SARS-CoV-2 PT (gray), SARS-CoV-2 D614G (pink), CX1 (green) and BANAL-20-52 (violet). The proteins are presented as cartoon. (**B**) Alignment of S2 subunit of S proteins from CoVs indicated above the panels. The RMSDs are shown under the alignment.

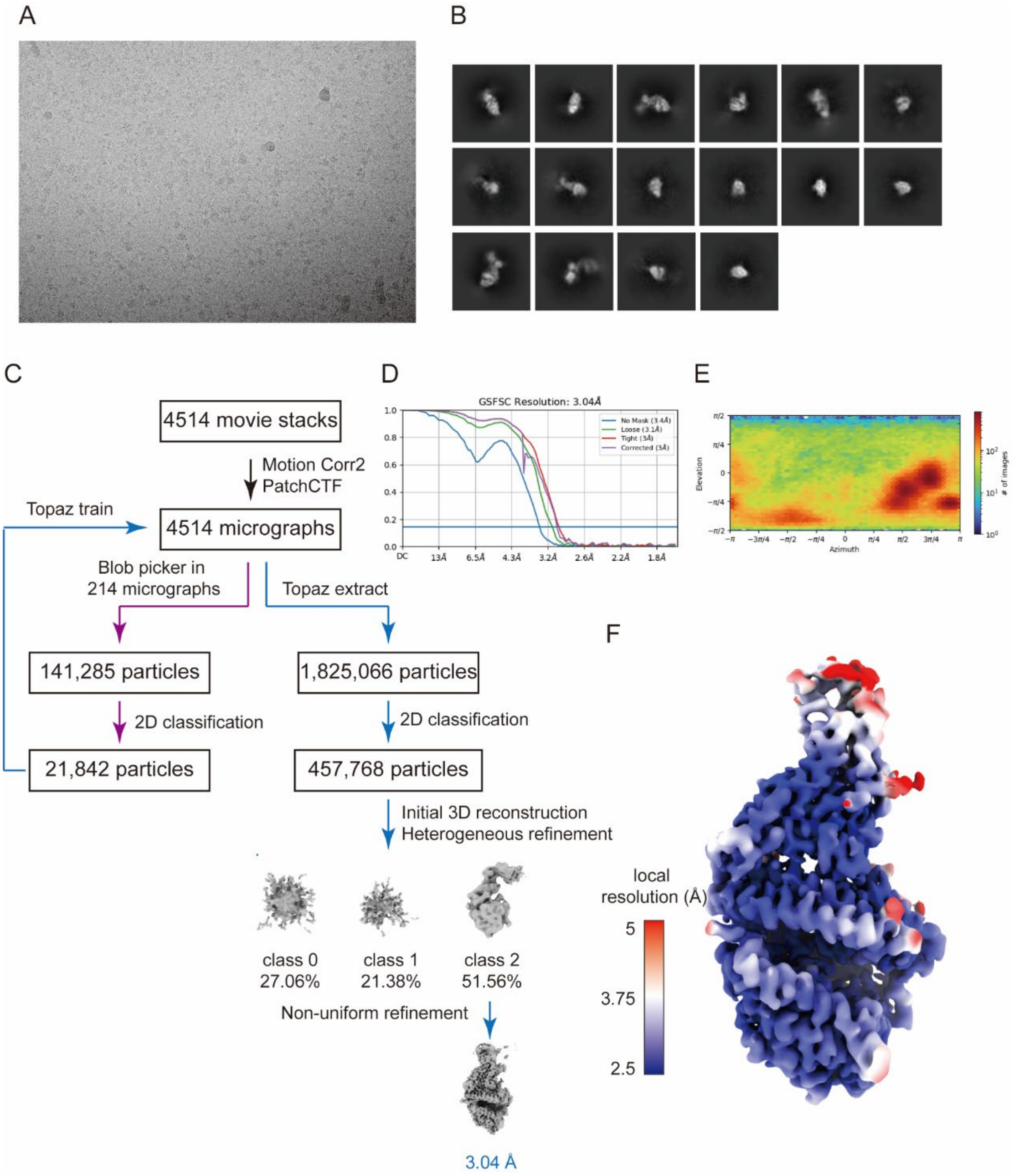

Fig. S9 Cryo-EM data processing of the CX1-RBD/hACE2 protein. (**A**) A representative electron micrograph. (**B**) 2D classes selected for reconstruction. (**C**) Main steps of image processing. (**D**) Local resolution estimation of the final volume. (**E**) Angular distribution of the particles. (**F**) Resolution distribution of the cryo-EM map, where blue represents high resolution areas, and red represents low resolution areas.

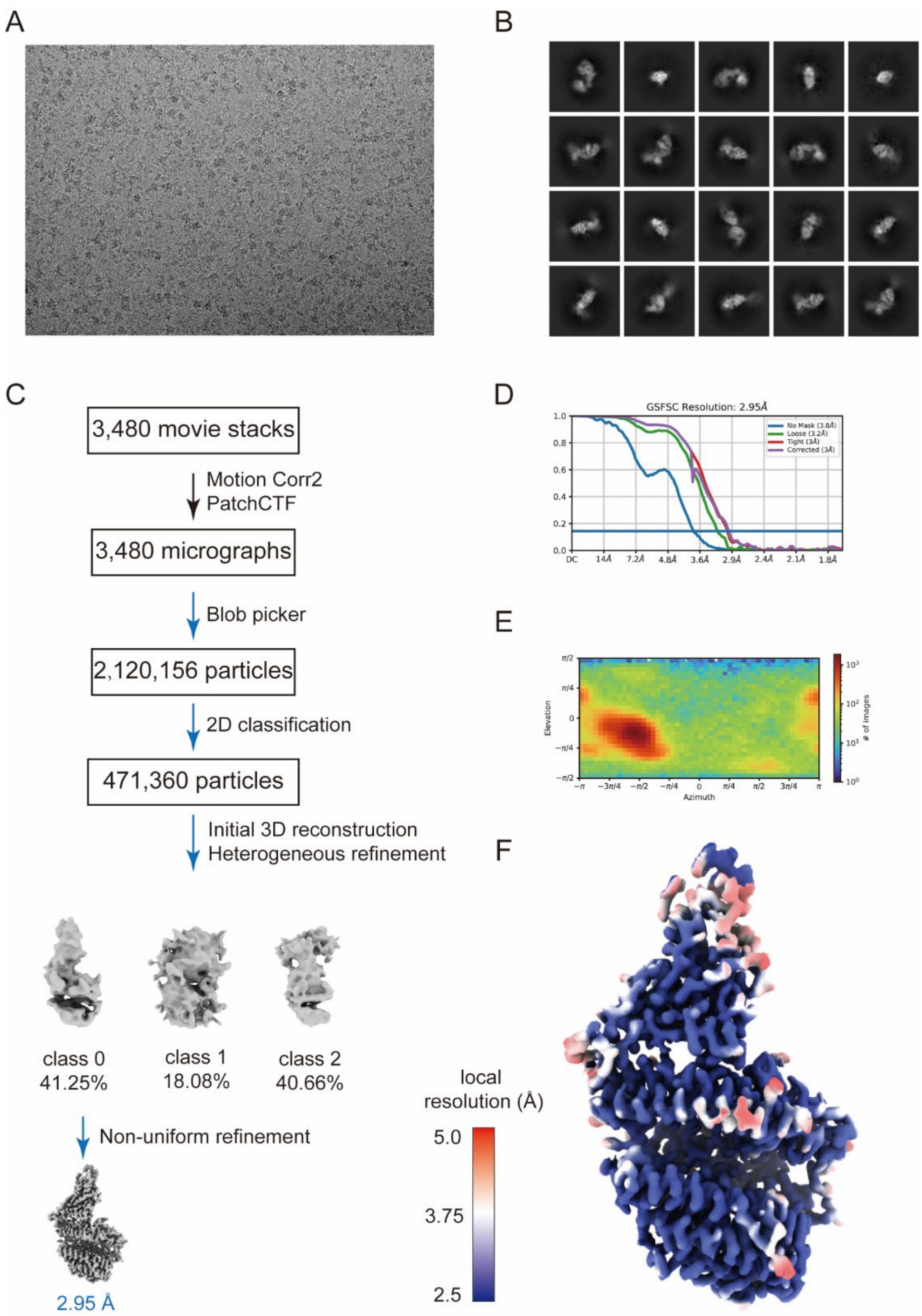

Fig. S10 Cryo-EM data processing of the BANAL-20-52-RBD/hACE2 protein. (**A**) A representative electron micrograph. (**B**) 2D classes selected for reconstruction. (**C**) Main steps of image processing. (**D**) Local resolution estimation of the final volume. (**E**) Angular distribution of the particles. (**F**) Resolution distribution of the cryo-EM map, where blue represents high resolution areas, and red represents low resolution areas.

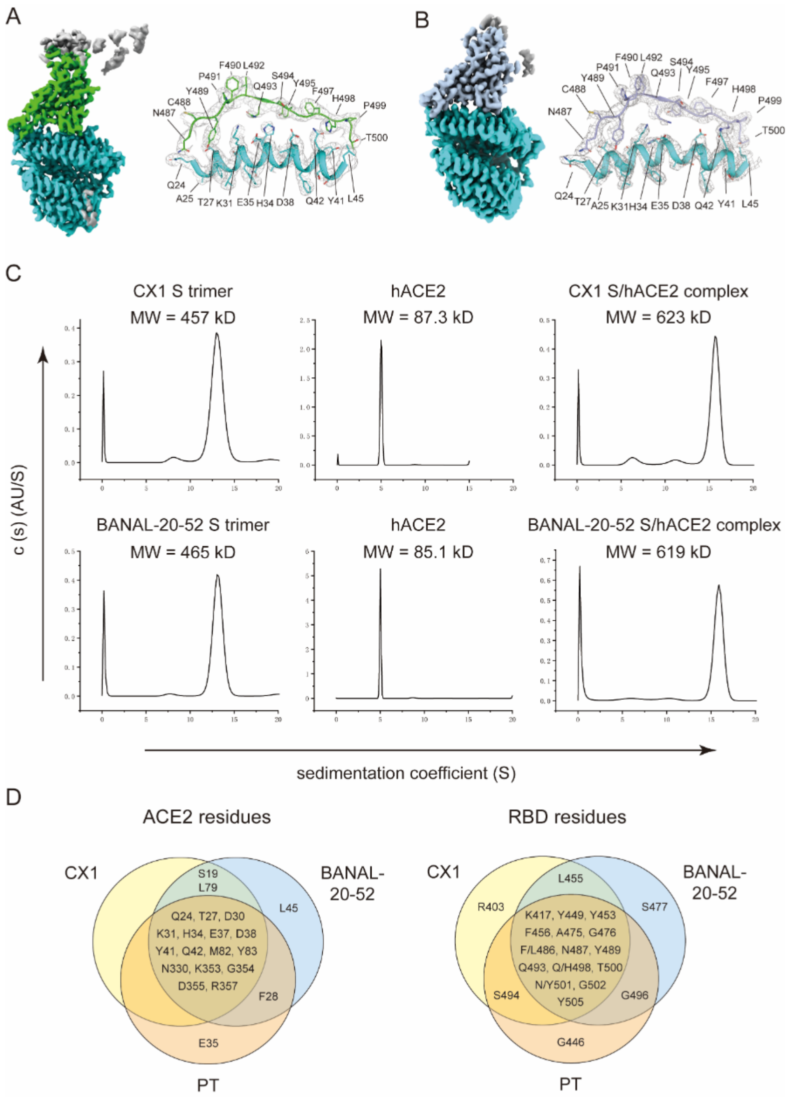

Fig. S11 Structure of CX1 S/hACE2 and BANAL-20-52 S/hACE2 complexes. (**A**) Cryo-EM map of CX1-RBD complexed with hACE2 (left) and interacting residues (right). The map is colored according to the constructed model and those parts without fitted model are colored in gray. (**B**) Cryo-EM map of BANAL-20-52-RBD complexed with hACE2 (left) and interacting residues (right). The map is colored according to the constructed model and those parts without fitted model are colored in gray. (**C**) Analytical ultracentrifugation absorbance data analysis of S trimer, hACE2 and S/hACE2 complexes. The calculated molecular weight is labeled. (**D**) Venn diagram of interacting residues of CX1, BANAL-20-52 and SARS-CoV-2 PT-RBD binding with hACE2.

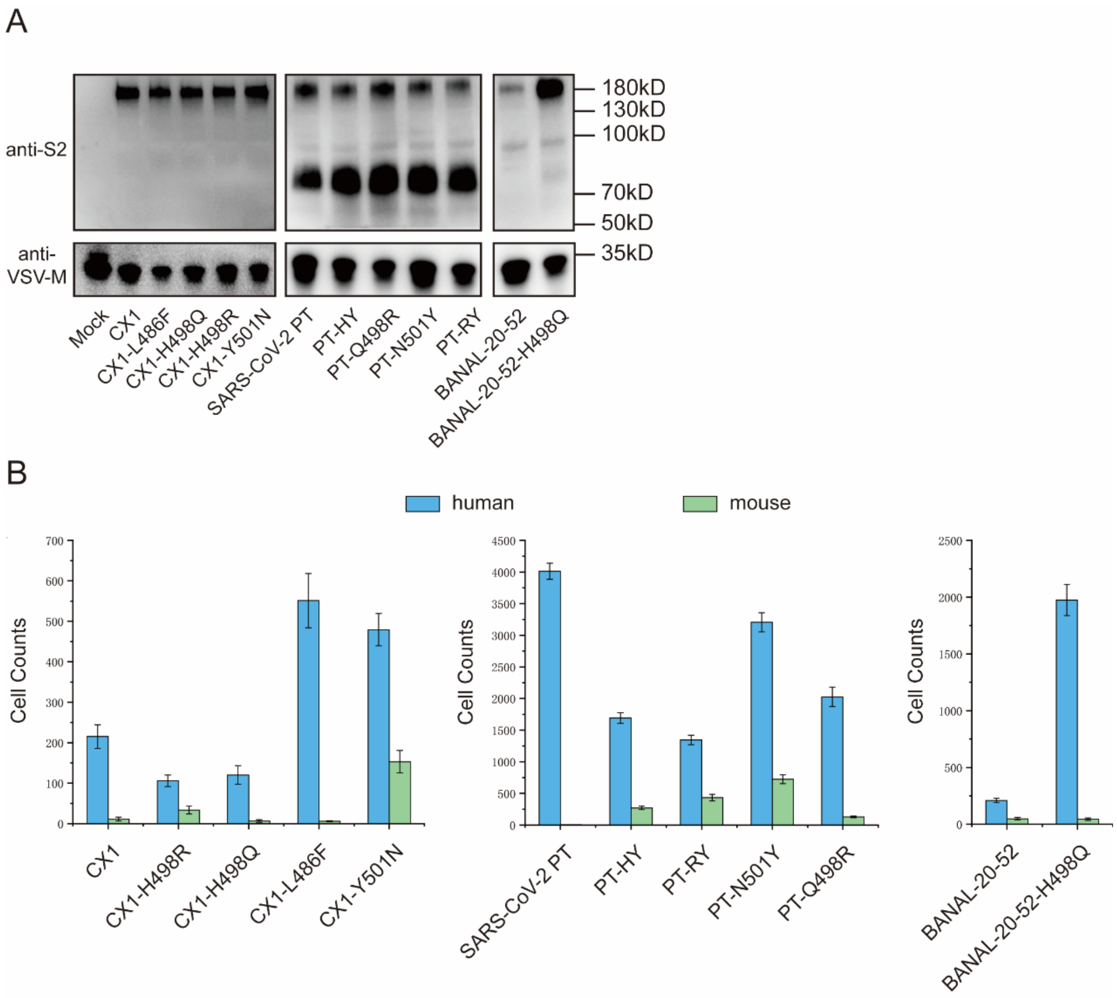

Fig. S12 Pseudovirus assay on the SARS-CoV-2, CX1 and BANAL-20-52 wildtype and variants. (**A**) Detection of S proteins of SARS-CoV-2, BANAL-20-52, CX1 and variants in pseudovirions by western blot assay. The S proteins are detected by anti-S2 polyclonal antibodies. VSV-M served as loading controls. (**B**) Pseudovirus assay of SARS-CoV-2, CX1 and BANAL-20-52 wildtype and variants. The assay was conducted on BHK-21 cells transiently transfected with human or mouse ACE2. Data were presented as mean ± SD. Two independent experiments were performed with similar results.

Table S1. Cryo-EM data collection, processing and refinement statistics.

	CX1-S	BANAL-20-52-S	CX1-RBD/hACE2	BANAL-20-52-RBD/hACE2
**Data collection and processing**				
Microscope	Titan Krios G3i	Titan Krios G3i	Titan Krios G3i	Titan Krios G3i
Magnification	105k	105k	105k	105k
Voltage (kV)	300	300	300	300
Electron exposure ($e^-/Å^2$)	60	60	60	60
Defocus range (μm)	-1.0 ~ -2.0	-1.0 ~ -2.0	-1.0 ~ -2.0	-1.0 ~ -2.0
Pixel size (Å)	0.69	0.69	0.69	0.69
Symmetry imposed	C1	C1	C1	C1
Final particle images (no.)	129,596	253,926	236,030	194,436
Map resolution (Å)	2.74Å	2.50Å	3.04 Å	2.95 Å
FSC threshold	0.143	0.143	0.143	0.143
**Refinement**				
Initial model used (PDB code)	6ZGI	6ZGI	6LZG	6LZG
Non-hydrogen atoms	25,979	25,878	6,369	6,488
Protein residues	3,236	3,219	771	789
Validation				
Clash score	6.61	5.62	17.54	7.83
Poor rotamers	0	0.14	1.47	0.29
R.m.s. deviations				
Bond length (Å)	0.005	0.005	0.004	0.007
Bond angles (°)	0.932	1.026	0.764	0.910
Ramachandran statistics (%)				
Most favored	90.74	93.22	95.82	94.78
Allowed	8.80	6.62	4.18	4.97
Disallowed	0.47	0.16	0.00	0.25
